# Deep multi-omics integration by learning correlation-maximizing representation identifies prognostically stratified cancer subtypes

**DOI:** 10.1093/bioadv/vbad075

**Published:** 2023-06-21

**Authors:** Yanrong Ji, Pratik Dutta, Ramana Davuluri

**Affiliations:** Division of Health and Biomedical Informatics, Department of Preventive Medicine, Northwestern University Feinberg School of Medicine, Chicago, IL 60611, USA; Department of Biomedical Informatics, Stony Brook Cancer Center, Stony Brook Medicine, Stony Brook University, Stony Brook, NY 11794, USA; Department of Biomedical Informatics, Stony Brook Cancer Center, Stony Brook Medicine, Stony Brook University, Stony Brook, NY 11794, USA

## Abstract

**Motivation:**

Molecular subtyping by integrative modeling of multi-omics and clinical data can help the identification of robust and clinically actionable disease subgroups; an essential step in developing precision medicine approaches.

**Results:**

We developed a novel outcome-guided molecular subgrouping framework, called Deep Multi-Omics Integrative Subtyping by Maximizing Correlation (DeepMOIS-MC), for integrative learning from multi-omics data by maximizing correlation between all input -omics views. DeepMOIS-MC consists of two parts: clustering and classification. In the clustering part, the preprocessed high-dimensional multi-omics views are input into two-layer fully connected neural networks. The outputs of individual networks are subjected to Generalized Canonical Correlation Analysis loss to learn the shared representation. Next, the learned representation is filtered by a regression model to select features that are related to a covariate clinical variable, for example, a survival/outcome. The filtered features are used for clustering to determine the optimal cluster assignments. In the classification stage, the original feature matrix of one of the -omics view is scaled and discretized based on equal frequency binning, and then subjected to feature selection using RandomForest. Using these selected features, classification models (for example, XGBoost model) are built to predict the molecular subgroups that were identified at clustering stage. We applied DeepMOIS-MC on lung and liver cancers, using TCGA datasets. In comparative analysis, we found that DeepMOIS-MC outperformed traditional approaches in patient stratification. Finally, we validated the robustness and generalizability of the classification models on independent datasets. We anticipate that the DeepMOIS-MC can be adopted to many multi-omics integrative analyses tasks.

**Availability and implementation:**

Source codes for PyTorch implementation of DGCCA and other DeepMOIS-MC modules are available at GitHub (https://github.com/duttaprat/DeepMOIS-MC).

**Supplementary information:**

Supplementary data are available at *Bioinformatics Advances* online.

## 1 Introduction

Advances in high-throughput technologies have facilitated generation of multiple -omics datasets, such as genomic, epigenomic, transcriptomic and proteomic measures, on same biosamples. Indeed, analyses of -omics data on hundreds of cancer tissues, profiled by The Cancer Genome Atlas (TCGA), International Cancer Genome Consortium (ICGC) and many other laboratories around the world, have led to the identification of clinically relevant subgroups of various cancers. While majority of the studies utilized microarray or RNA-Seq-based expression profiling data (Alizadeh *et al.*, 2000; Golub *et al.*, 1999; Guinney *et al.*, 2015; Pal *et al.*, 2014; Shilpi *et al.*, 2019; Verhaak *et al.*, 2010), several others have employed other -omics data, such as DNA methylation (Figueroa *et al.*, 2010; Ma *et al.*, 2020; Stefansson *et al.*, 2015), copy number variation (Curtis *et al.*, 2012; Taylor *et al.*, 2010) and microRNA (Bhattacharyya *et al.*, 2015; Sokilde *et al.*, 2019). Nevertheless, great inconsistency on the number and assignment of subjects/samples to different subgroups was often observed between different molecular subtyping studies utilizing data from different -omics types, and sometimes even data from same platforms, lowering the reproducibility of such research (Zhao *et al.*, 2019).

While single -omic analyses have produced valuable insights, integrative analyses of multiple -omics datasets can lead to improved cancer subtyping into clinically relevant subgroups and potentially identify cancer biomarkers or predictors of cancer progression, under the assumption that different -omics types represent different biological processes and complement each other in reducing noises and revealing better insights related to biology of the tumor (Rappoport and Shamir, 2018). Subtypes that show good concordance across different -omics studies should capture the consensus information and interdependencies between multiple -omics datasets and therefore could be more robust, reproducible and information-rich than those that are derived by the analyses of single -omics datasets. It was indeed also proven that such integrative approaches often lead to enhanced predictions on patient prognosis (Wang *et al.*, 2014). To this end, many multi-omics integration methods have been proposed and successfully employed for cancer subtyping. Early integration methods attempt to merge the original feature matrices from different -omics levels prior to clustering, which typically handles the high dimensionality via regularization and feature selection (Wu *et al.*, 2015). In contrast, late integration methods perform single -omics clustering on individual -omics data first and then attempt to integrate the clustering solutions in some way, allowing more flexibility on choice of clustering algorithms (Nguyen *et al.*, 2017). Other intermediate integration methods, such as similarity-based (Wang *et al.*, 2014), generalized matrix factorization-based (Zhang *et al.*, 2012), and Bayesian statistical modeling-based ones (Mo *et al.*, 2018), are also present and widely used.

In recent years, deep learning has emerged to become the state-of-the-art model in various fields and tasks, including image classification, speech recognition, language modeling and many others (LeCun *et al.*, 2015). It has also been successfully applied on diverse Bioinformatics tasks and achieved superior performance (Min *et al.*, 2017). From a deep multi-view learning perspective, different -omics levels can be seen as individual views, or modalities, collected on same data, while the objective is to learn a shared nonlinear representation that simultaneously encodes all modalities by preserving maximal information through deep neural networks (Wang *et al.*, 2015). To date, majority of deep learning-based multi-omics integrative subtyping methods use autoencoders for learning of such representations by either early or intermediate integration. Using autoencoder, Chaudhary et al integrated gene expression, methylation and miRNA expression by finding a latent representation that best reconstructs the concatenated original feature matrix containing three -omics levels and discovered statistically significant prognostic difference among liver cancer patients (Chaudhary *et al.*, 2018). The use of autoencoders was later extended to other types of cancers (Poirion *et al.*, 2018; Zhang *et al.*, 2018) with more advanced autoencoder architectures being employed (Simidjievski *et al.*, 2019; Zhang *et al.*, 2019). However, standard autoencoder-based dimensionality reduction often does not account for the correlation or interrelationship between views, as the objective only typically seeks to minimize the reconstruction error independently for each view. In contrast, canonical correlation analysis (CCA)-based approaches attempt to find shared representation that maximizes the correlation between views and therefore can capture common features shared across -omics. As a result, CCA-based approaches are often found to outperform autoencoder-based models in multi-view learning (Wang *et al.*, 2015).

In this work, we present DeepMOIS-MC (Deep Multi-Omics Integrative Subtyping by Maximizing Correlation), a novel deep learning-based method that achieves multi-omics integration and subtyping of cancer by finding a low-dimensional shared representation that maximizes the correlation between multiple views. DeepMOIS-MC extends DGCCA (Deep Generalized Canonical Correlation Analysis) (Benton *et al.*, 2019), a canonical correlation analysis-based algorithm that can simultaneously learn nonlinear relationships between more than two views. The hypothesis is that the shared embedded space that maximizes correlation between views should contain the most useful information for robust subtyping, since this indicates that certain patterns are repeatedly seen across multiple -omics platforms. We show that DeepMOIS-MC is indeed capable of robustly and accurately identifying cancer subtypes with enhanced prognostic stratification that are translatable across platforms.

## 2 Methods

### 2.1 Datasets, study design and preprocessing

We obtained TCGA level-3 datasets from UCSC Xena database (https://xena.ucsc.edu/) using the R package *UCSCXenaTools* (Goldman *et al.*, 2020; Wang and Liu, 2019). Three types of -omics data from four types of cancer (LIHC, hepatocellular carcinoma, *n* = 438; BRCA, breast invasive carcinoma, *n* = 1233 and LUAD, lung adenocarcinoma, *n* = 617) were obtained in total, including RNA-seq (UNC IlluminaHiSeq_RNASeqV2), miRNA-seq (BCGSC IlluminaHiSeq_miRNASeq) and methylation array (JHU-USC HumanMethylation450) data. Data was preprocessed in a similar fashion to previous work (Chaudhary *et al.*, 2018). Namely, for DNA methylation data, only methylation array probes labeled within 200 and 1500 bp upstream of transcription start site (TSS200 and TSS1500) were kept with their methylation β-value averaged, which becomes the averaged β-value for the corresponding CpG island. To handle missing values, any feature that contains more than 20% missing value or zeros will be discarded, while any sample with more than 20% missing value or zeros will be removed as well. For the rest of the features and samples, K-nearest neighbor will be used for imputation as implemented in R *impute* package if there are any remaining missing values. Since the DGCCA algorithm supports the handling of missing views for samples, all samples instead of only those that contains all three types of -omics data were kept. For the confirmation cohorts, we accessed ICGC portal and downloaded RNA-seq data from LIRI-JP cohort, which is a Japanese population of hepatocellular carcinoma (HCC) patients (*n* = 243) (Fujimoto *et al.*, 2016). The expression values were log transformed to make it commensurate with TCGA expression values. We also downloaded GSE14520 from the Gene Expression Omnibus (GEO) database (Clough and Barrett, 2016) which contains gene expression microarray data from Affymetrix GeneChip HG-U133A platform for *n* = 225 HCC patients (Roessler *et al.*, 2010).

### 2.2 Overview of DeepMOIS-MC

#### 2.2.1 DeepMOIS-MC pipeline

**Figure 1.**
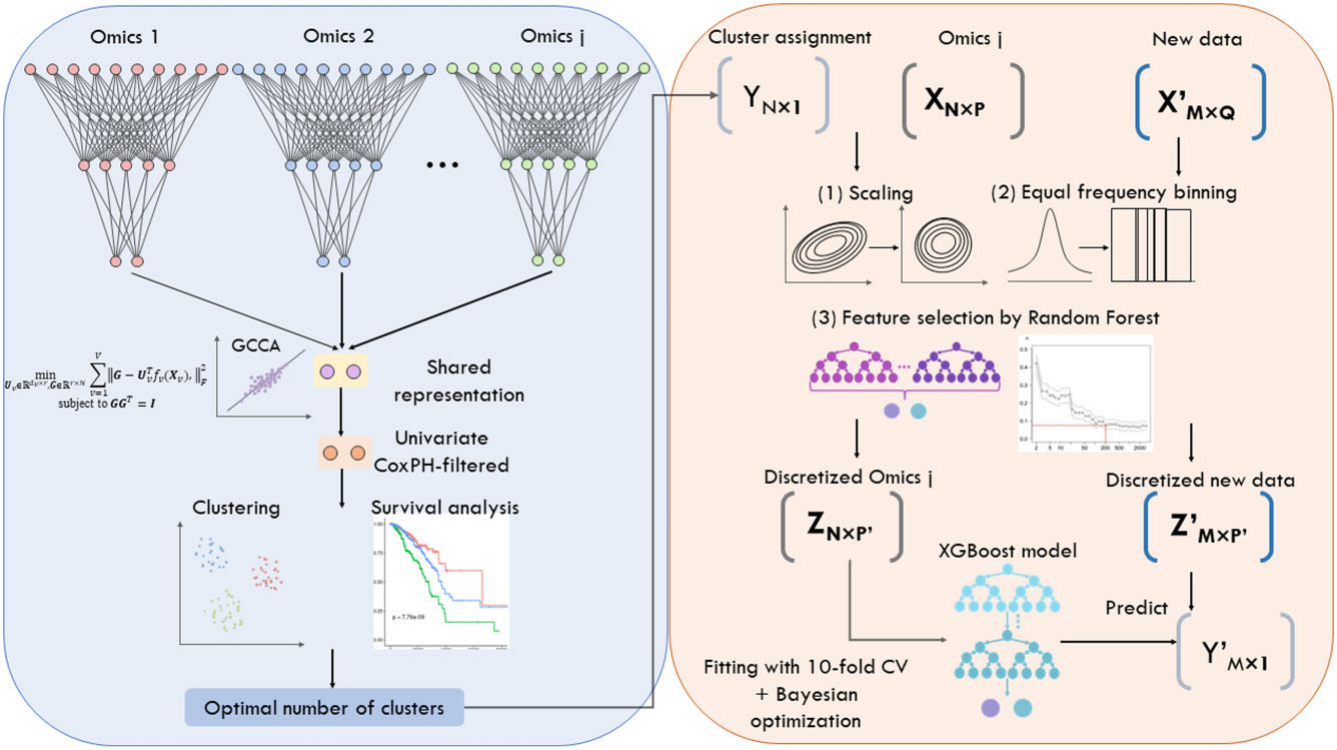
Schematic workflow of the DeepMOIS-MC subtyping pipeline. *X* and *X*’ indicate the original training data and new data for -omics *j* respectively, while *Z* and *Z*’ indicate the discretized version of the matrices. *Y* and *Y*’ indicate clustering labels for the training and new data. *N*, number of samples in *X*; *M*, number of samples in *X*’; *P*, number of features in *X*; *Q*, number of features in *X*’; *P*’, number of (common) features after feature selection

DeepMOIS-MC (Fig. 1) is divided into two stages: clustering (left) and classification (right). In the clustering stage, the preprocessed high-dimensional j -omics views were input into two-layer fully connected neural networks. The outputs of individual networks were subjected to Generalized Canonical Correlation Analysis (GCCA) loss to learn the shared representation. Next, the learned representation was filtered by univariate Cox proportional hazard (Cox-PH) model to select survival-related features. The filtered features were used for clustering and Kaplan–Meier survival analysis to determine the optimal cluster assignments. In the classification stage, the original feature matrix for j-th -omics view was scaled and discretized based on equal frequency binning, and then subjected to feature selection using random forest. This results in a discretized feature matrix with reduced dimensions. Using these features, an XGBoost model was built with 10-fold cross-validation. The trained model will then be used for prediction of new incoming data, which was similarly preprocessed and discretized as the training data.

#### 2.2.2 Deep generalized canonical correlation analysis

Canonical correlation analysis (CCA) (Hotelling, 1936) finds linear projections between two random vectors ${(x}_{1},\;x_{2})\in R^{n_{1}}\times R^{n_{2}}$, such that the maximally correlated projection directions are: where $\Sigma_{11}$ and $\Sigma_{12}$ are covariance matrices of $x_{1}$ and $x_{2}$, while $\Sigma_{12}$ is cross-covariance matrix between them. The projections are constrained to have unit variance. In general, if we have V different views of data, a generalized form of CCA (GCCA) (Horst, 1961) can be expressed as the following optimization problem: where $X_{v}\in R^{d_{v}\times N}\;$is the data from vth view, $d_{v}$ is the dimension of vth view, N is the total number of data points, $U_{v}$ is the projection vector of vth view, and $G\in R^{r\times N}\;$is the combined representation of multiple views we aim at learning. Although GCCA allows derivation of shared representation of more than two views, the transformation itself (i.e. $U_{v}^{T}X_{v}$) is still restricted to be linear, which is a major limitation of both approaches. Recently, DGCCA method was proposed for multi-view representation learning. DGCCA extends deep canonical correlation analysis (DCCA) (Andrew *et al.*, 2013), by combining the flexibility of nonlinear (deep) representation learning with the statistical power of incorporating information from many independent sources. A formal definition of DGCCA network is analogous to GCCA, except for the linear transformation $U_{v}^{T}X_{v}$ being substituted as $U_{v}^{T}{f_{v}(X}_{v})$, where $f_{v}\;$represents the neural network architecture for the v-th view, while ${f_{v}(X}_{v})$ denotes the output of final layer of $f_{v}$. The objective thus becomes


$${(\omega }_{1}^{*},\omega_{2}^{*})=\text{arg}\underset{\omega_{1},\omega_{2}}{\text{max}}\mathrm{corr}(\omega_{1}^{T}x_{1},\;\omega_{2}^{T}x_{2})=\text{arg}\underset{\omega_{1},\omega_{2}}{\text{max}}\frac{\omega_{1}^{T}\Sigma_{12}\omega_{2}}{\sqrt{\omega_{1}^{T}\Sigma_{11}\omega_{1}\omega_{2}^{T}\Sigma_{22}\omega_{2}}}=\arg\underset{\omega_{1}^{T}\Sigma_{11}\omega_{1}{=\omega }_{2}^{T}\Sigma_{22}\omega_{2}=1\;}{\max}\omega_{1}^{T}\Sigma_{12}\omega_{2},$$

$$\underset{U_{v}\in R^{d_{v}\times r},\;G\in R^{r\times N}\;}{\min}\sum_{v=1}^{V}{\left\|G-U_{v}^{T}X_{v}\right\|}_{F}^{2}\;\mathrm{subject}\;\mathrm{to}\;GG^{T}=I,$$

$$\underset{U_{v}\in R^{d_{v}\times r},\;G\in R^{r\times N}\;}{\min}\sum_{v=1}^{V}{\left\|G-U_{v}^{T}{f_{v}(X}_{v}),\;\right\|}_{F}^{2}\;\mathrm{subject}\;\mathrm{to}\;GG^{T}=I$$

The final shared representation G obtained should be an r×N dimensional nonlinear manifold that indicates the maximum correlation between all modalities.

### 2.3 Implementation of DGCCA algorithm

We have adapted the original implementation of DGCCA with necessary modifications to ensure all functions can operate properly (Benton *et al.*, 2019). To ensure consistency, we used 2-layer feedforward networks with 500 nodes for hidden layer and 100 nodes for the final output embedding layer and *ReLU* activation function throughout all views (Supplementary Table S1). To prevent overfitting, we added an L1-regularization with constant 0.001 and an L2-regularization with constant 0.0001 to all network weights. We set both the dimensionality of embedding and the rank of low-rank approximation to the view matrices to be 100, consistent with the number of nodes in the output layer. We also added a regularization of 10^−6^ to each view’s covariance matrix and assigned equal DGCCA weights between all views. The optimization was done using gradient descent with 20 epochs to train the DGCCA model. Since PyTorch is more widely used, we have also reimplemented the DGCCA algorithm in PyTorch as an independent package to facilitate the broader use by the community.

### 2.4 Outcome-guided clustering

The DGCCA algorithm outputs a 100-dimensional vector embedding, which reflects the nonlinear maximally correlated shared representation. These embeddings were then subjected to a univariate Cox-PH feature selection process prior to clustering. Essentially, each coordinate of the 100-dimensional embedding was used as a feature and input to a univariate Cox-PH model, and only features with significant log-rank *P*-value (*P* < 0.05) were preserved. The selected features were then used as input to K-means clustering with 100 initialization points and 2000 iterations, where the best number of clusters (*k*) was determined by combination of visual analysis of the silhouette plot, the average silhouette width and the Calinski–Harabasz score. We applied log-rank test to determine the statistical significance of prognostic stratification of the subtypes derived. We plotted Kaplan–Meier survival curves and performed Cox-PH analysis using R *survival* and *survminer* packages.

We benchmarked DeepMOIS-MC with several most widely used multi-omics integration and subtyping methods, including iClusterBayes (Mo *et al.*, 2018), LRACluster (Wu *et al.*, 2015), SNF (Wang *et al.*, 2014) and PINSPlus (Nguyen *et al.*, 2019), using the CEPICS platform in R (version 3.6.0) (Duan *et al.*, 2019). We followed Scenario 3 in the CEPICS guide and computed a patient–patient similarity matrix based on the embeddings as input to the package. We set max number of clusters to be six and did not upload the true labels since they are not available, so the evaluation of clustering was done in an internal way. As all the other algorithms require explicit filtering of samples that are missing in one or more -omics (whereas DeepMOIS-MC handles data points with missing views internally), we only compared DeepMOIS-MC with other algorithms on the subset of data where all views were present, which resulted in slight performance differences. We used default parameters for all built-in methods implemented in CEPICS and selected the top 10% of features in median absolute deviation (MAD) for each view as inputs to the four methods, following the default setting in the original CEPICS paper.

### 2.5 Feature selection, classification and validation

In order to identify multi-omics biomarkers associated with the obtained subtypes, and to achieve platform-independent prediction of subtypes on future data, we extended the classification stage of our previous PIGExClass algorithm to build three independent classifiers for gene expression, methylation and miRNA data respectively (Pal *et al.*, 2014; Shilpi *et al.*, 2019). To achieve platform transition (i.e. two platforms of the same -omics, e.g. microarray versus RNA-seq, or two array platforms), we first normalized individual -omics view in our training data by median scaling, then applied equal frequency data discretization to transform each scaled feature into ranked bins as we have done previously (Pal *et al.*, 2014). Next, for each view, we selected the optimal number of discretized features by recursive feature elimination with 10-fold cross-validation with 10 repeats using Random Forest. The optimal number of features will be used to build a XGBoost classifier to predict on subtypes of future samples using *scikit-learn* API to *xgboost* module in Python (Chen and Guestrin, 2016). We performed hyperparameter tuning using Bayesian optimization using Python module *skopt* with 10-fold cross validation.

To confirm the robustness of derived subtypes, we validated the results on two independent confirmation cohorts. We began by finding common features between the confirmation cohort and our training data of corresponding -omics in TCGA, followed by the classification steps illustrated above. Note that the feature selection and training of classifier were only done using the overlapping features. To further eliminate the platform and batch differences between the training and confirmation datasets, we standardized the confirmation datasets using the mean and standard deviation of the training set, following median scaling and before data discretization. For discretization, instead of re-binning the confirmation datasets themselves, we placed every validation sample into the bins formed by the training data, so that the validation samples are consistent and comparable with the training samples in scale. We used R package *recipes* to predict the bins of training data that validation samples belonged to.

## 3 Results

### 3.1 DeepMOIS-MC identifies two differential subtypes in hepatocellular carcinoma patients

To evaluate the ability of DeepMOIS-MC to extract significant survival-associated subtypes, we first obtained TCGA-LIHC data for 438 patients with hepatocellular carcinoma (HCC). Among the 438 patients, 406 contains all three types of -omics data (gene expression by RNA-seq, DNA methylation array and miRNA-seq) while 22 has at least one type of -omics data missing. Contrary to other similar studies (Chaudhary *et al.*, 2018), we performed subtyping on all 438 patients without removing those with missing data, since DGCCA supports input of data points with missing views. After preprocessing, we obtained 19 832 genes whose promoter region (1500 bp upstream of TSS) mapped to CpG probes for DNA methylation data, 15 587 genes for RNA-seq gene expression data and 261 miRNAs from miRNA-seq data. Instead of stacking the three feature matrices and input into a single network, we fed the three views of data into three independent 2-layer feedforward neural networks and used the outputs of the three networks to compute GCCA loss (Fig. 1).

**Figure 2.**
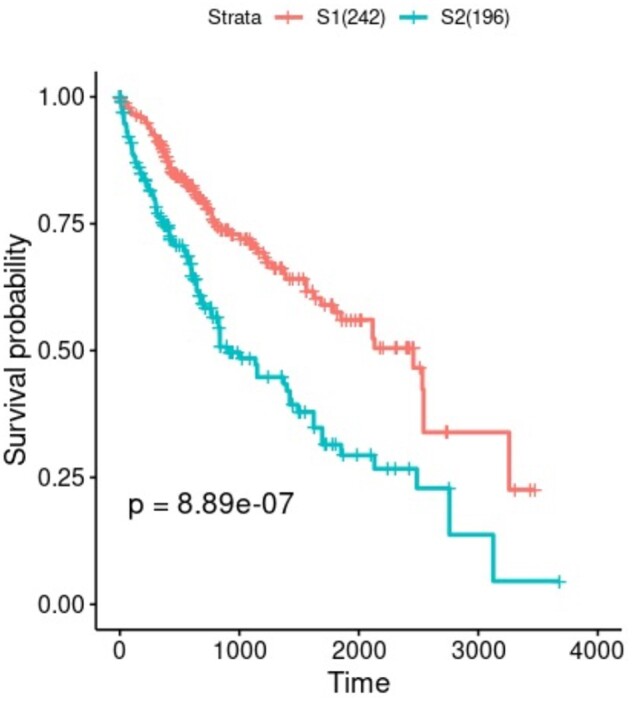
Kaplan–Meier survival curves for the two subtypes of TCGA-LIHC

After deriving a 100-dimensional representation that best summarizes all three views, we applied univariate Cox-PH analysis for selection of survival associated features (log-rank *P* < 0.05). Upon univariate Cox-PH filtering, eight variables were retained for k-means clustering analysis with cluster number from two to six. The silhouette index shows both *k* = 2 and 6 could be the optimal number of k (Supplementary Fig. S1c). However, Calinski–Harabasz score shows a uniformly decreasing trend from *k* = 2 to 6, indicating *k* = 2 should be a better choice. Meanwhile, upon further inspection of the silhouette plots, we noticed that for *k* = 6 one of the clusters (cluster 5) has a lot of samples with negative silhouette width (Supplementary Fig. S2) whereas *k* = 2 results in two near perfectly separated clusters (Supplementary Fig. S1a). As such, we eventually selected *k* = 2 to be the best number of clusters. We further visualized the clusters and confirmed that the two clusters can be roughly separated even on 2D subspace spanned by the first two CoxPH filtered DGCCA embedding coordinates or on the first two principal components (Supplementary Figs S1b and S3). Furthermore, Kaplan–Meier survival analysis shows that the two clusters form two subtypes that are very significantly different in overall survival (log-rank *P* = 8.89e–7; Fig. 2). We therefore conclude that DeepMOIS-MC is capable of extracting HCC patient subgroups that are prognostically distinct.

### 3.2 DGCCA outperforms autoencoders in learning survival-associated representations

Since most deep learning-based multi-omics integrative subtyping methods utilize variants of autoencoders, we decided to compare DGCCA’s performance on extracting survival-associated representations with that of autoencoders. We then trained several simple variants of autoencoders commonly used in literatures, including (i) stacked autoencoder with concatenated inputs, i.e. to combine input views and analyze through a single neural network (AE); (ii) stacked autoencoder with separated input views, processed through separated neural networks, then concatenated the latent representation/embedding (AE_concat); (iii) denoising autoencoder (dropout at input layer to corrupt some input) with concatenated inputs (DAE); (iv) denoising autoencoder with concatenated embedding (DAE_concat); (v) denoising autoencoder, concatenated inputs with additional dropout at every hidden layer (DAE_full_dropout); (vi) denoising autoencoder, concatenated embedding with additional dropout at every hidden layer (DAE_full_dropout_concat); (vii) variational autoencoder, concatenated inputs (VAE); and (viii) variational autoencoder, concatenated embedding (VAE_concat). To ensure consistency with previous literatures and with DGCCA, we used same network architecture for the fully connected layers (one hidden layer with 500 nodes in the encoder, 100 nodes for the bottleneck layer and one hidden layer with 500 nodes in the decoder for stacked and denoising autoencoders; for variational autoencoders, same structure applies, except that a sampling layer/variational block used to replace the bottleneck layer) throughout all experiments. We also used hyperbolic tangent (tanh) as activation function as this was most widely used in previous studies for autoencoders (Chaudhary *et al.*, 2018; Poirion *et al.*, 2018; Zhang *et al.*, 2018). The optimization was done using stochastic gradient descent with learning rates chosen such that the validation loss dropped steadily and smoothly. For most experiments, this learning rate was found to be 10^−2^, while for variational autoencoders the learning rate was set at 10^−5^ to avoid gradient explosion. We trained autoencoders in all 8 settings for 20 epochs. To ensure the models were not overfitting, we employed an early stopping criterion with a patience of 3 epochs, while we also compared the results at 10 epochs versus at 20 epochs, since many existing methods train autoencoders for 10 epochs. All other steps, including univariate Cox-PH analysis, k-means clustering and survival analysis, also remained exactly same as DGCCA. In all experiments, the best numbers of clusters happened to be *k* = 2 as determined again by silhouette analysis and Calinski–Harabasz score.

We generated Kaplan–Meier survival curves for the 2 identified HCC subtypes for all 8 autoencoder settings at both 10 and 20 epochs (Supplementary Fig. S4). Despite ensuring steady decrease of loss throughout the 20 epochs in every experiment, i.e. the latent autoencoder representation obtained at epoch 20 should theoretically be the best in reconstructing inputs, we observed that the subtypes representing most significantly different survival subgroups were not guaranteed to be obtained at the end of training. In most cases, representations that led to significant survival difference were instead obtained at epoch 10. Meanwhile, there seemed to be around 15–19 outliers outside of the large cluster, and autoencoders were prone to only capture this simple feature if improperly trained. Further, the denoising autoencoders with separated inputs but concatenated embeddings, whether adding dropout after every layer or not (DAE_concat or DAE_full_dropout_concat), did not seem to work well in this case. Among all experiments, the most significant *P*-value was obtained by denoising autoencoder with 50% dropout at every layer (DAE_full_dropout) at 10 epochs (log-rank *P* = 9.58e–5), which we believe was closest to the reported implementation and results by Chaudhary et al (Chaudhary *et al.*, 2018), since we cannot reproduce the exact original *P*-value due to the difference in number of TCGA-LIHC samples we used (438 versus 360). Nevertheless, this is still much less significant than the subtypes identified using DGCCA representation (*P* = 8.89e–7) we obtained at the end of 20-epoch training.

### 3.3 DeepMOIS-MC outperforms traditional approaches in patient stratification

**Figure 3.**
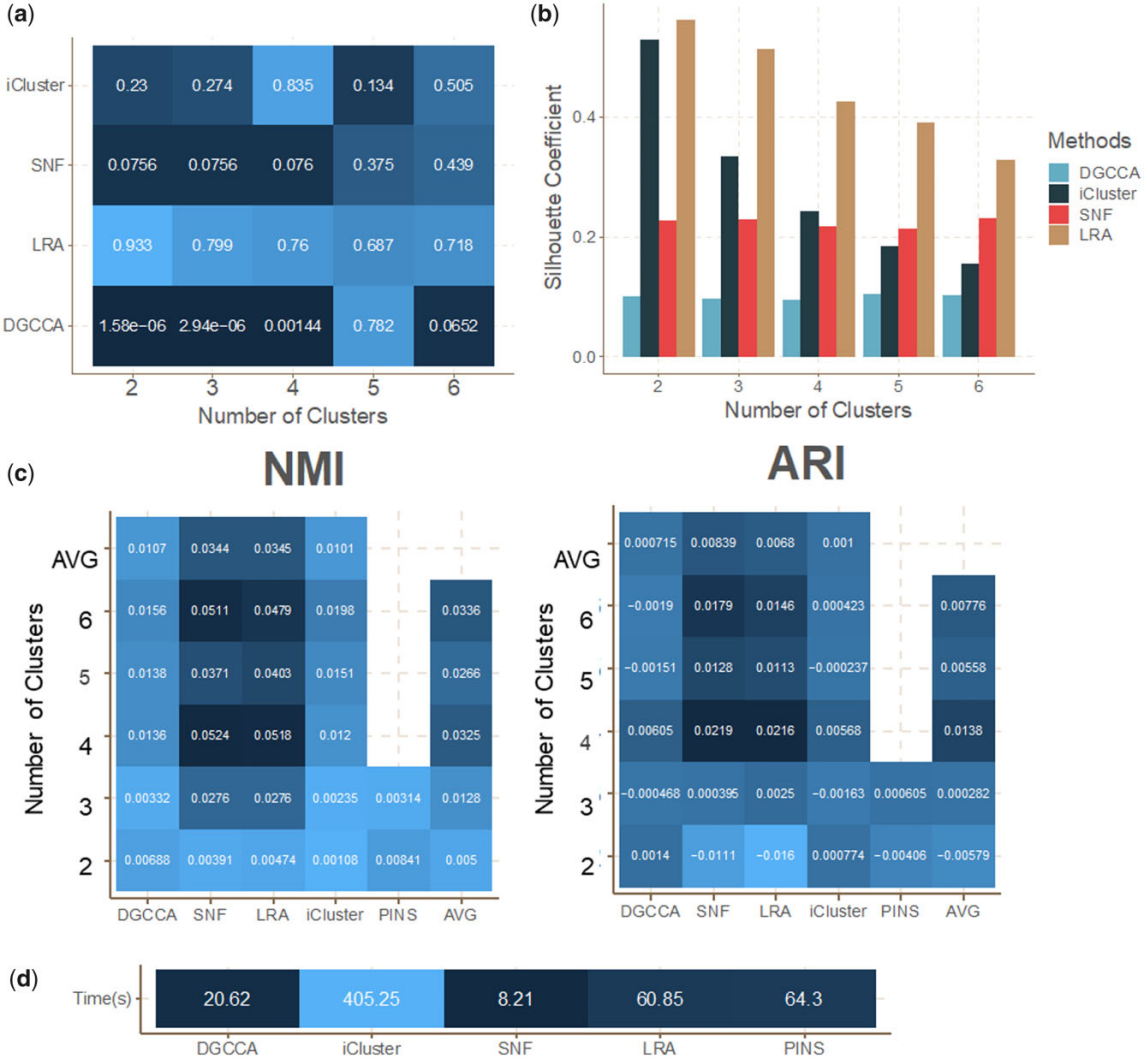
CEPICS output for comparison of DeepMOIS-MC (DGCCA) with four traditional multi-omics integrative subtyping methods on TCGA-LIHC data. (**a**) Cox-PH *P*-values of survival difference among subtypes identified. (**b**) Silhouette coefficients of different methods for various numbers of clusters. (**c**) Average of normalized mutual information (NMI) and adjusted rand index (ARI) between every pair of methods at different number of clusters. (**d**) Time consumption of each methods

Having evaluated the quality of DGCCA-generated representation in extracting survival-associated HCC subtypes as compared to autoencoders, we further compared DeepMOIS-MC, which uses DGCCA-generated representations, with four traditional multi-omics integrative subtyping tools that were most used in the community using CEPICS platform (Methods). DeepMOIS-MC (labeled DGCCA) was the only method that resulted in significant Cox *P*-value (*P* < 0.05) in *k* = 2–6 among all methods, including PINSPlus (*P* = 0.82206 for *k* = 2 and *P* = 0.16087 for *k* = 3; Supplementary Fig. S5) which was not included in the figure (Fig. 3a). Although DeepMOIS-MC had much lower silhouette coefficients compared to the other three methods (iClusterBayes, SNF, LRA; Fig. 3b), the silhouette coefficients between DeepMOIS-MC and the other methods should not be directly comparable since they were computed on completely different data (univariate Cox-PH-filtered embedding for DGCCA versus top 10% of features ranked by MAD for other methods). Thus, the silhouette coefficients were only useful to determine the optimal number of clusters k here within the same method. The average normalized mutual information (NMI) and adjusted rand index (ARI) basically indicate that the subgroups identified by DeepMOIS-MC was dissimilar to those found by all other methods (Fig. 3c). Finally, we compared the time consumption of DeepMOIS-MC with other methods, since deep learning approaches were often criticized for being time-consuming. DeepMOIS-MC took less than 30 s to identify the subtypes with a full-batch training for 20 epochs, which was the second best among all five methods (Fig. 3d).

**Figure 4.**
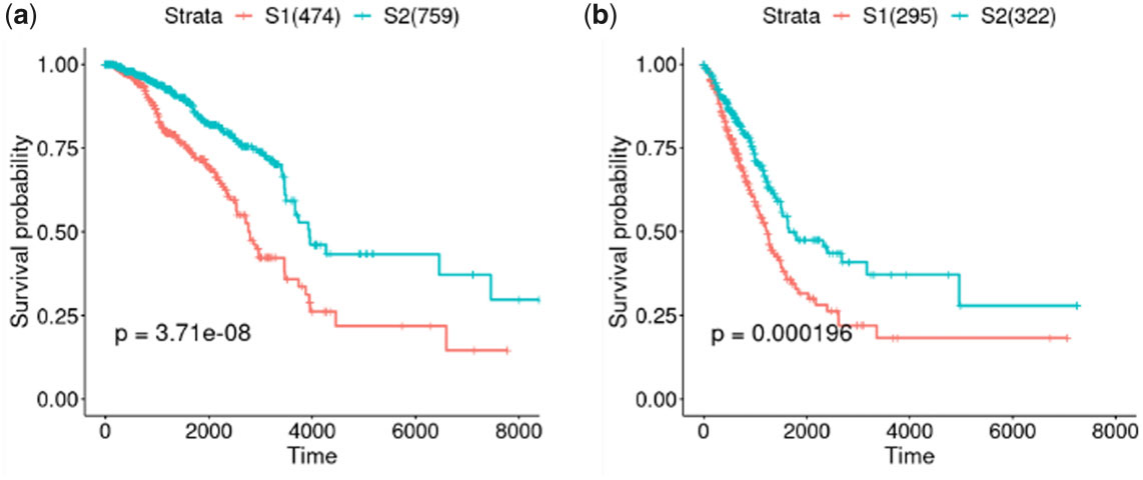
Kaplan–Meier survival curves for two identified subtypes of (**a**) BRCA and (**b**) LUAD

We further extended our comparison to the other two types of cancer, including breast adenocarcinoma (BRCA) and lung adenocarcinoma (LUAD) in TCGA. Even without altering any training parameters and network architecture, DeepMOIS-MC consistently identified two subtypes in both types of cancer that were significantly separated in survival (log-rank *P* = 3.71e–8 for BRCA and *P* = 1.96e–4 for LUAD; Fig. 4), while other numbers of clusters led to even more significant subgrouping as well (Supplementary Fig. S6). We then again performed comparisons using CEPICS, and both comparisons confirmed that DeepMOIS-MC outperformed traditional methods in obtaining patient subgroups significantly associated with survival (Supplementary Figs S7 and S8).

### 3.4 Subtypes identified are robustly validated across cohorts and translated across platforms

**Figure 5.**
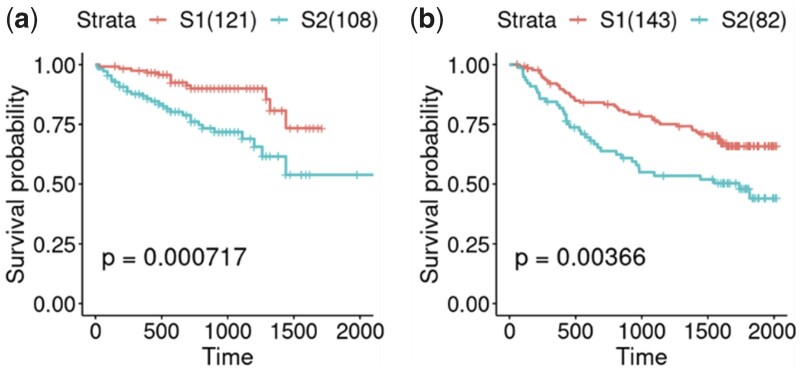
Kaplan–Meier survival curves for two identified subtypes of (**a**) ICGC LIRI-JP cohort and (**b**) GSE14520 cohort

We finally tested whether the subtypes we identified could be validated in independent cohorts and translated from one platform to another using DeepMOIS-MC. To achieve this, we accessed and downloaded two independent HCC datasets from ICGC (LIRI-JP cohort) and GEO (GSE14520), the first being on RNA-seq platform and second being a gene expression microarray dataset. 15 189 and 10 125 genes out of 15 588 in TCGA-LIHC training set were found in the two validation sets respectively. To eliminate the noise related to platform bias and batch effects, we applied equal frequency data discretization on both training and validation data after scaling (Section 2). The number of bins was a hyperparameter to choose and was determined by whether finer or coarser binning enhanced performance of the model (Pal *et al.*, 2014). For the two validation cohorts (ICGC and GSE14520), we determined the optimal number of bins to be 30 and 15 respectively. We selected the best number of features via random forest recursive feature elimination with 10-fold cross validation (CV) with 10 repeats. In both cases, the optimal number of features was determined near the ‘elbow’ region of the feature selection plot, where the curve just started to reach plateau, to prevent overfitting. The features used were chosen to be top 150 and 190 for ICGC and GSE14520 respectively (Supplementary Fig. S9). We used the selected features to build XGBoost models with 10-fold CV. The ICGC LIRI-JP model with 150 vars and 30 bins achieved cross-validation area under ROC curve (AUC) of 0.8838 and accuracy of 0.7995, while the GSE14520 model with 190 vars and 15 bins attained AUC of 0.8571 and accuracy of 0.7615. Kaplan–Meier analysis showed that both stratifications have resulted in statistically significant survival differences (*P* = 7.17e–4 and 3.66e–3 respectively; Fig. 5), indicating that the classification models implemented as part of DeepMOIS-MC were robust in picking up the survival-associated subtypes initially identified in TCGA-LIHC cohort. As the GSE14520 was a gene expression microarray dataset, the result also demonstrated that the platform transition was successfully achieved.

### 3.5 DeepMOIS-MC can be extensible to include other -omics data

Finally, we examined whether DeepMOIS-MC can be readily extensible to additional -omics data by bringing new modality in the DGCCA stage. Since genomic alterations are another source of heterogeneity across patient samples and were frequently used in subtyping studies in the past, we decided to include copy number variation (CNV) as a fourth view and investigate how that could change our results. We have performed combinatorial experiments between the four types of -omics data and reported the subtypes extracted in each experiment on LIHC, BRCA and LUAD data (Supplementary Figs S10–S12). Though some other combinations also result in clinically relevant subtypes indicated by distinct survival, we confirmed that our original setting (mRNA expression + miRNA expression + DNA methylation) still resulted in most statistically significant subtypes. Nevertheless, this confirmed our method’s capability to readily incorporate additional -omics data across different platforms as new modalities.

## 4 Discussion

In this study, we proposed a novel deep learning-based multi-omics integrative cancer subtyping pipeline to derive a shared representation that simultaneously reconstructs the nonlinear transformations of all input views by maximizing the correlation across views. In theory, the biggest advantage of using a DGCCA-based representation as opposed to an autoencoder-based one is that it considers the relationship and attempts to extract only the common, coherent features among all views, rather than preserving and reconstructing all features of the views without explicitly considering how they are related to one another. Essentially, DGCCA finds the intersection of the nonlinear subspaces formed independently by the neural network outputs of all views (Sorensen *et al.*, 2021). This way, data on different -omics levels could serve as supporting evidence to each other, eliminating unwanted noises to a large extent. Ideally, if certain tumor samples exhibit some pattern on methylome level (e.g. they cluster together), it is also anticipated that a similar pattern would be observed on transcriptome level as well, while DGCCA could effectively identify such shared patterns. We hypothesized that these patterns could serve as better features on deriving the survival-associated latent subgroups among cancer patients. By direct comparing with various configurations of autoencoders, we confirmed that this hypothesis is true.

Although the -omics data introduced in this study included gene expression, DNA methylation, miRNA expression and copy number variation for benchmark purposes, we demonstrated that more types of -omics data (e.g. gene isoforms, reverse-phase protein array data, etc.) may also be simply integrated using DeepMOIS-MC to obtain potentially better subtypes with even more clinical value, given that DGCCA supports the input of an arbitrary number of views. Meanwhile, the fact that each -omics is processed through a separate network offers an additional advantage in network selection as different most suitable network architecture could be selected for individual view. Furthermore, our ability to incorporate data points with partially missing -omics also greatly extends the flexibility and applicability, especially when data is limited.

Notwithstanding, certain caveats exist in the current proposed model. First, although DGCCA specializes in extracting the common features across multiple views through maximizing correlation, the assumption that there are always some common patterns shared across the views is not guaranteed in every case. In a multi-omics scenario, if the quality of the training data for at least one platform is poor, there could be very weak consistency between the different -omics levels. In such cases, it is anticipated that DGCCA will not perform well since there is little or no common features to extract across views. Meanwhile, DGCCA could also be biased toward finding only common features across all views but ignoring some strong features that only present in single view. It is therefore necessary to balance the tradeoff between preserving single -omics features and finding common features shared across platforms. Moreover, the current study is limited by availability of public validation data for -omics other than expression that (i) simultaneously contains the -omics data and survival data, (ii) matches the exact platforms used in our training data and (iii) is not part of TCGA or its overlap with other consortiums, which will be addressed in future studies. Finally, as an outcome-guided subtyping approach, DeepMOIS-MC relies on high quality and comprehensiveness of survival data used as input for DGCCA model training to ensure generalizability of subtypes obtained across different cohorts.

## Supplementary Material

vbad075_Supplementary_Data
