# Supplementary material for: Deep multi-omics integration by learning correlation-maximizing representation identifies prognostically stratified cancer subtypes
Source: Bioinform Adv. 2023 Jun 21;3(1):vbad075. doi: 10.1093/bioadv/vbad075 (PMC10328436; doi:10.1093/bioadv/vbad075)
Supplement: vbad075_Supplementary_Data [file vbad075_supplementary_data.pdf]

Supplementary Materials
Deep Multi-Omics Integration by Learning Correlation-Maximizing Representation Identifies Prognostically Stratified Cancer Subtypes

Yanrong Ji, Pratik Dutta and Ramana V Davuluri[†1]

[1]Driskill Graduate Program in Life Sciences, Northwestern University, Chicago, IL,
[2]Department of Biomedical Informatics, Stony Brook Cancer Center, Stony Brook Medicine, Stony Brook University, Stony Brook, NY 11794
[†]Correspondence to Ramana.Davuluri@stonybrookmedicine.edu

Supplementary Figures

Figure S1 DeepMOIS-MC identifies two subtypes of TCGA-LIHC with DGCCA-learned representation. (a) Silhouette plot for k=2 clusters, red dotted line indicates average silhouette width (silhouette index). (b) Visualization of the clusters on first two DGCCA embeddings after Cox-PH filtering. (c) Silhouette width (index) and Calinski-Harabasz score across different number of clusters.

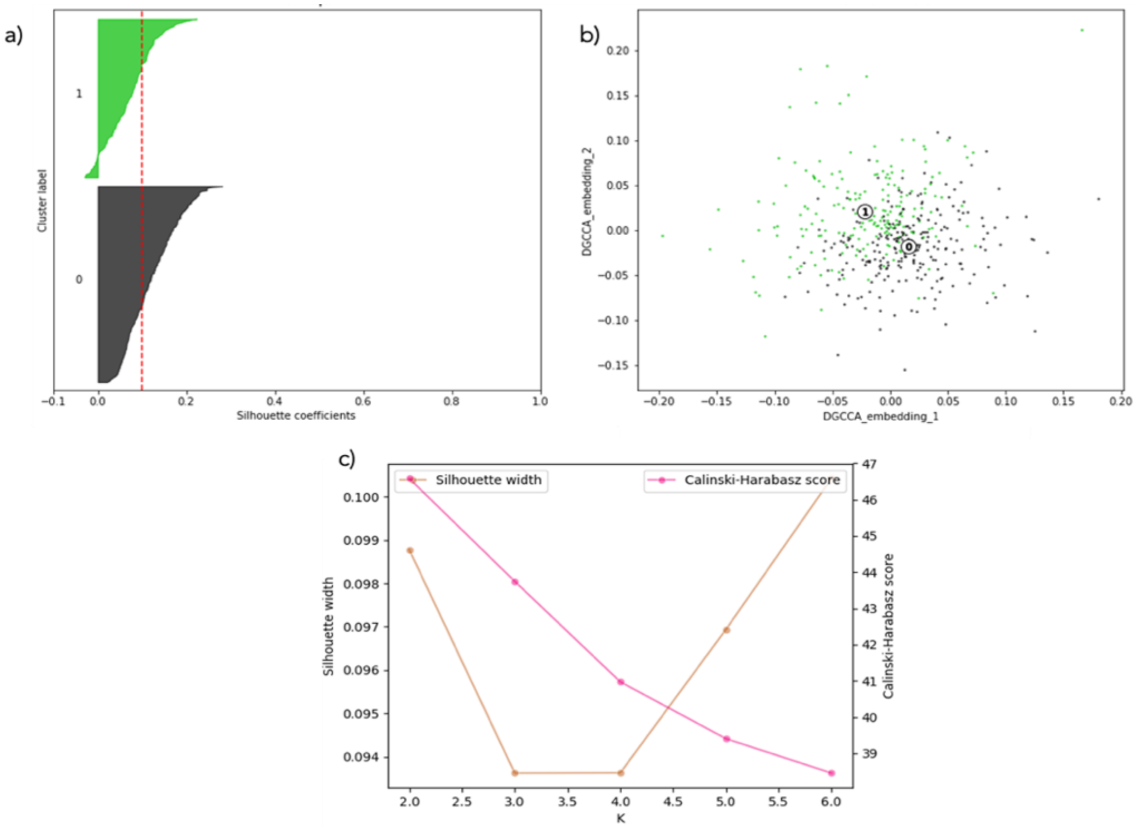

Figure S2 Silhouette plots for DeepMOIS-MC clustering on TCGA-LIHC samples (k=2 to 6).

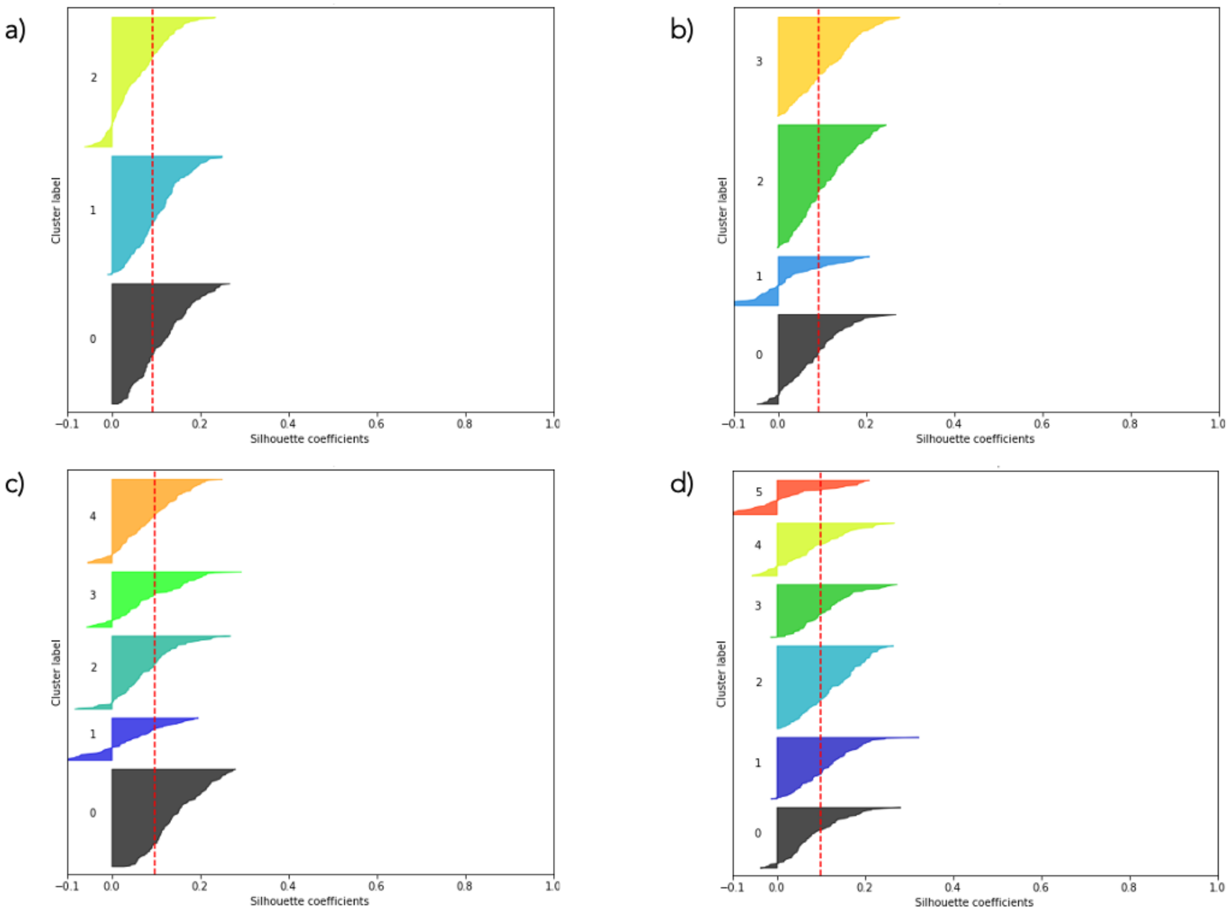

Figure S3 PCA plot of DGCCA embeddings for two identified subtypes of TCGA-LIHC data.

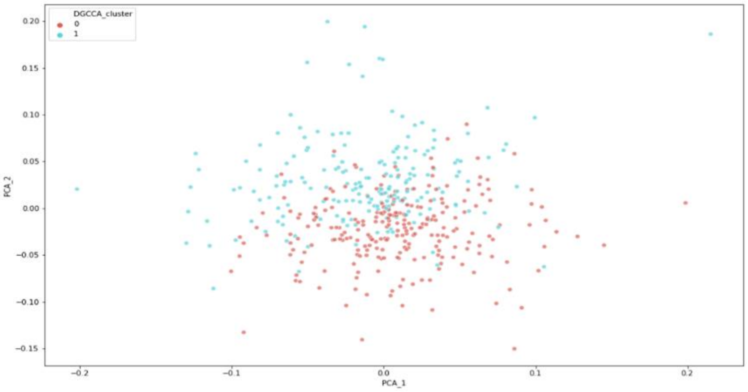

Figure S4. Kaplan-Meier survival curves for subtypes identified with different autoencoder implementations. All panels show two subplots with 10 epochs on the left and 20 epochs on the right. (a) simple (stacked) autoencoders with concatenated initial input matrix (AE); (b) simple (stacked) autoencoders with separate inputs and concatenated embedding (AE_concat); (c) denoising autoencoders with concatenated initial input matrix (DAE); (d) denoising autoencoders with separate inputs and concatenated embedding (DAE_concat); (e) denoising autoencoders with concatenated inputs and dropout after every hidden layer (DAE_full_dropout); (f) denoising autoencoders with concatenated embedding and dropout after every hidden layer (DAE_full_dropout_concat); (g) variational autoencoder with concatenated inputs (VAE); (h) variational autoencoder with concatenated embeddings (VAE_concat)..

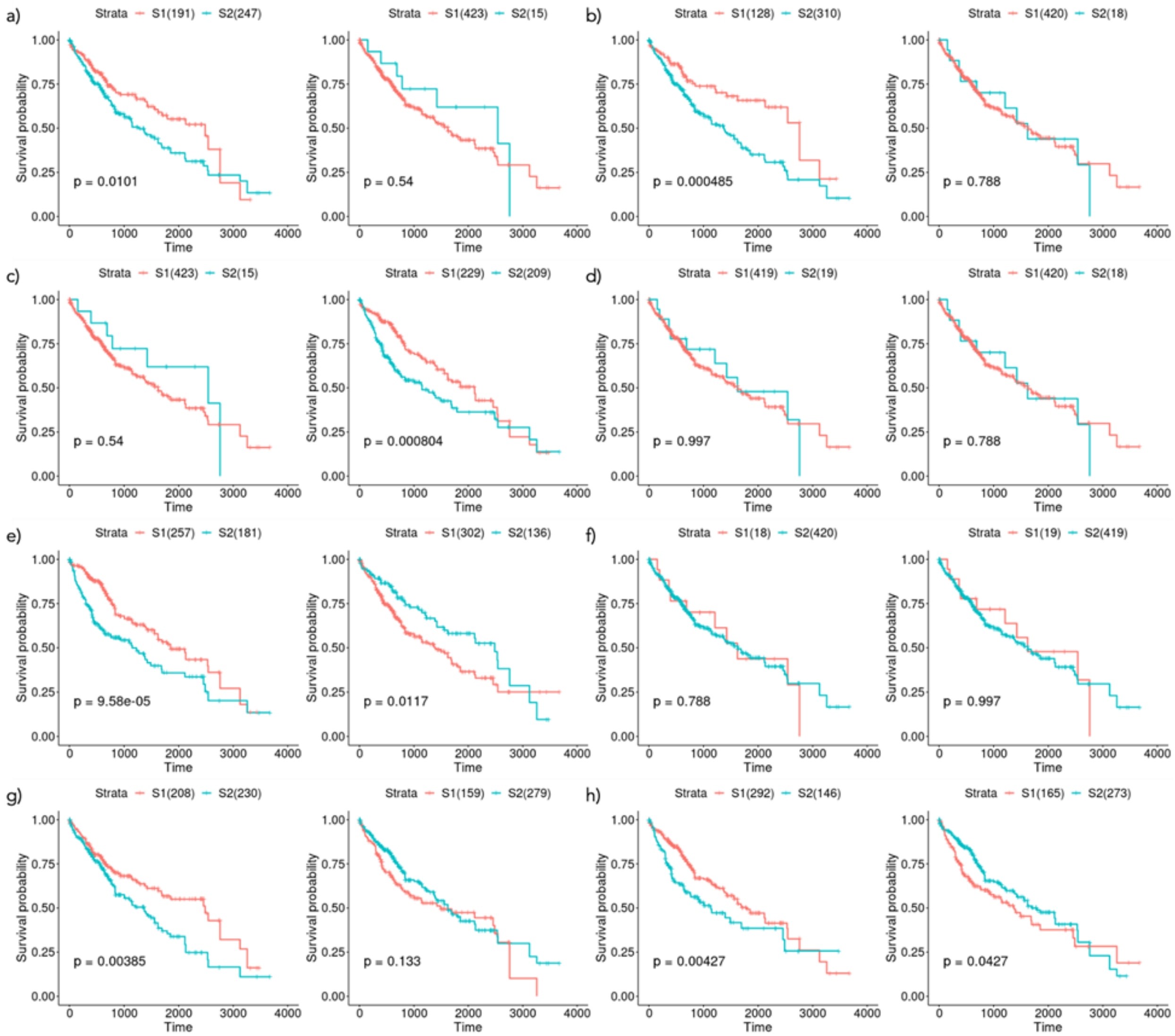

Figure S5 Kaplan-Meier survival curves for TCGA-LIHC subtypes identified using PINSPlus as implemented in CEPICS.

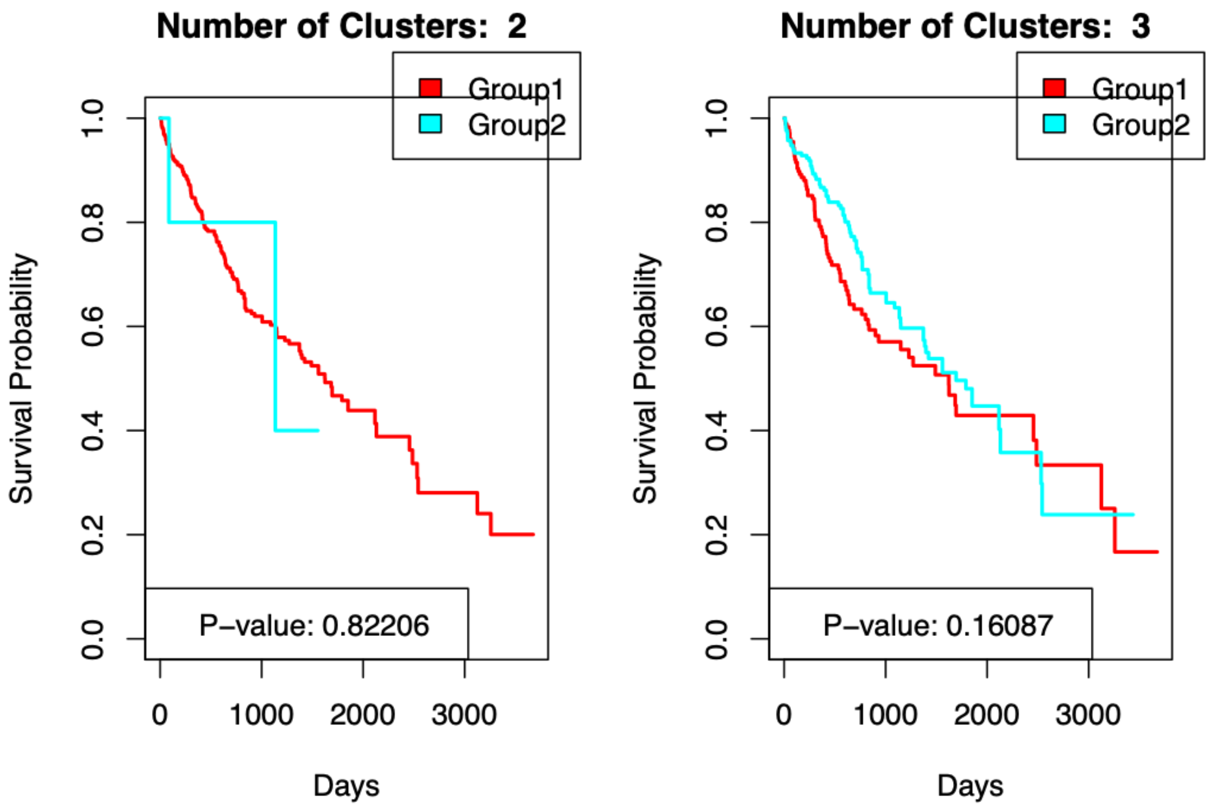

Figure S6: Kaplan-Meier survival curves for (a) BRCA and (b) LUAD subtypes identified using DeepMOIS-MC. (left to right) subtypes with k=3 to 6.

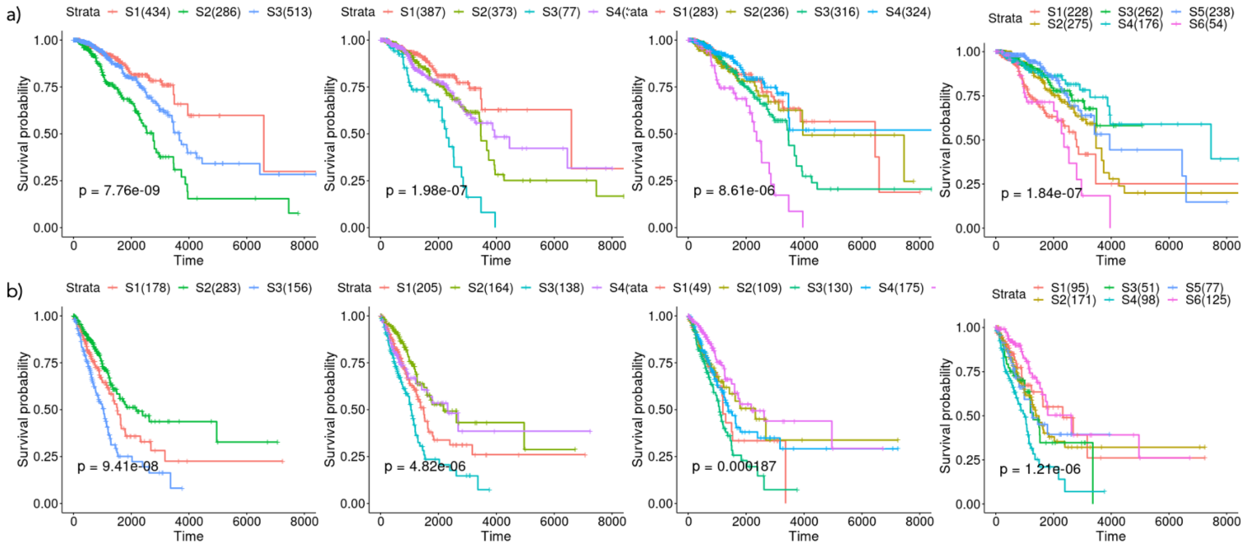

Figure S7 CEPICS output for comparison of DeepMOIS-MC (DGCCA) with four traditional multi-omics integrat ive subtyping methods on TCGA-BRCA data. (a) Cox-PH p-values of survival difference among subtypes identified. (b) Silhouette coefficients of different methods for various numbers of clusters. (c) Average of normalized mutual information (NMI) and adjusted rand index (ARI) between every pair of methods at different number of clusters. (d) Time consumption of each methods.

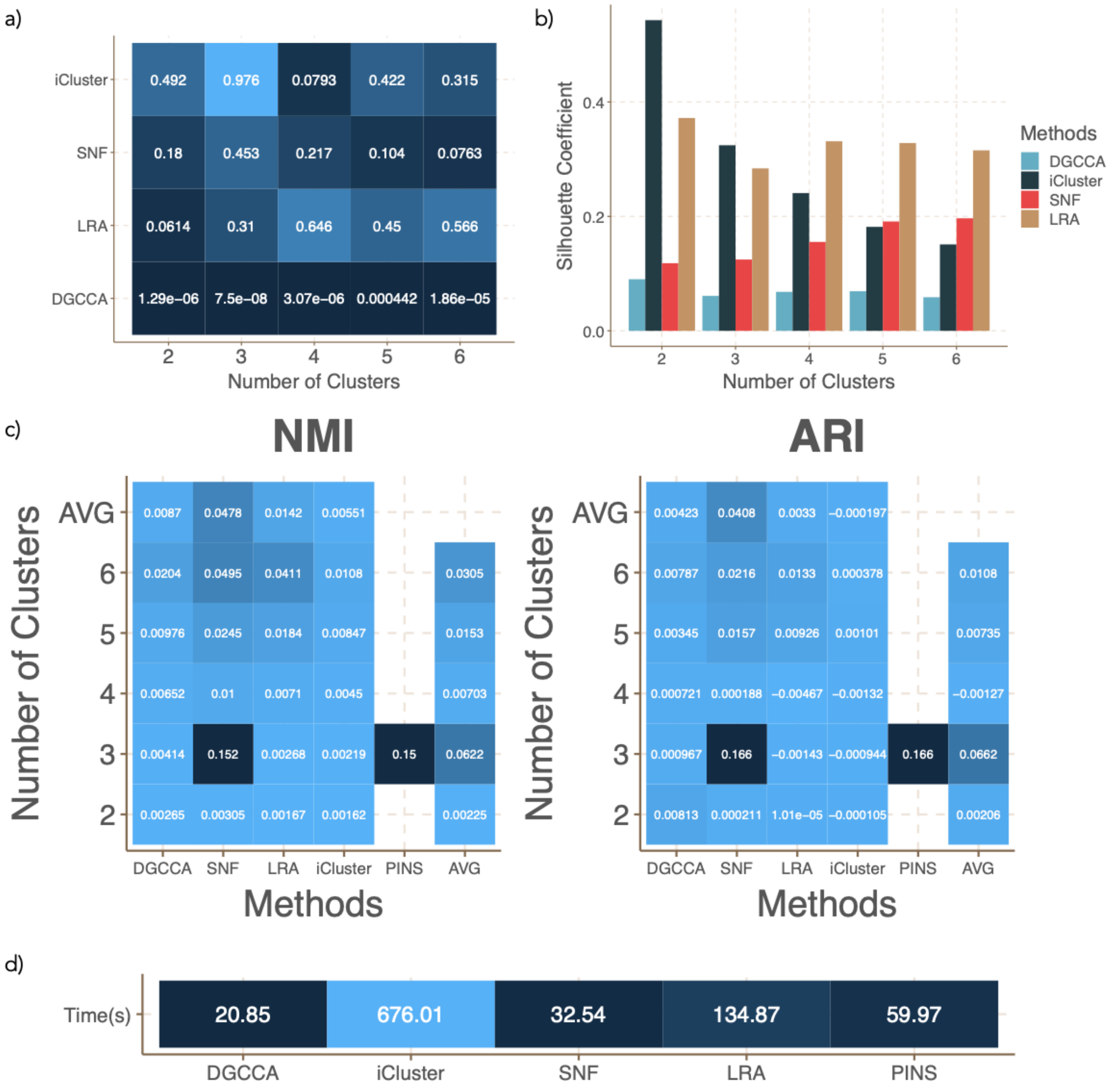

Figure S8 CEPICS output for comparison of DeepMOIS-MC (DGCCA) with four traditional multi-omics integrative subtyping methods on TCGA-LUAD data. (a) Cox-PH p-values of survival difference among subtypes identified. (b) Silhouette coefficients of different methods for various numbers of clusters. (c) Average of normalized mutual information (NMI) and adjusted rand index (ARI) between every pair of methods at different number of clusters. (d) Time consumption of each methods.

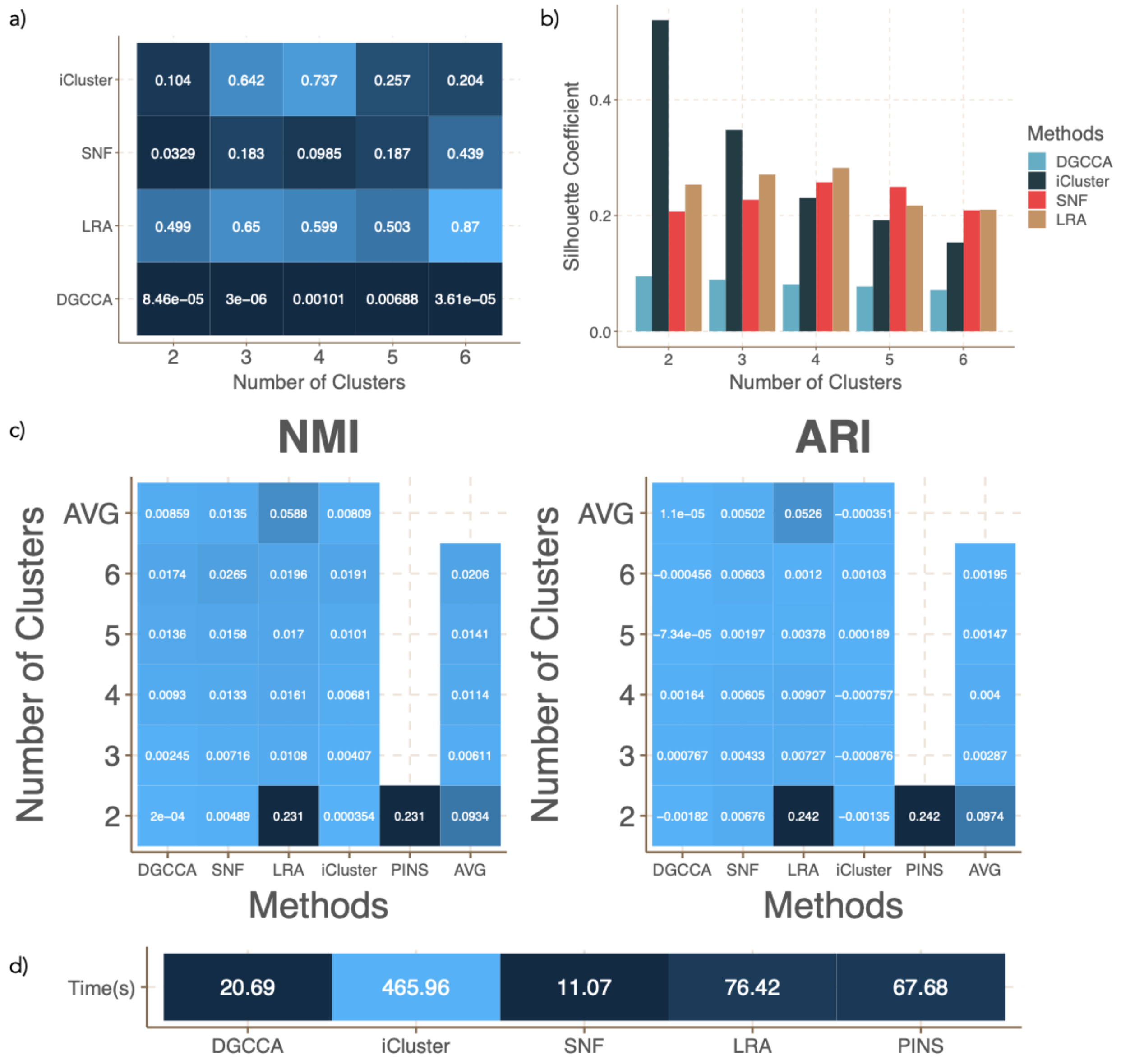

Figure S9 Feature selection plots for (a) ICGC LIRI-JP and (b) GSE14520.

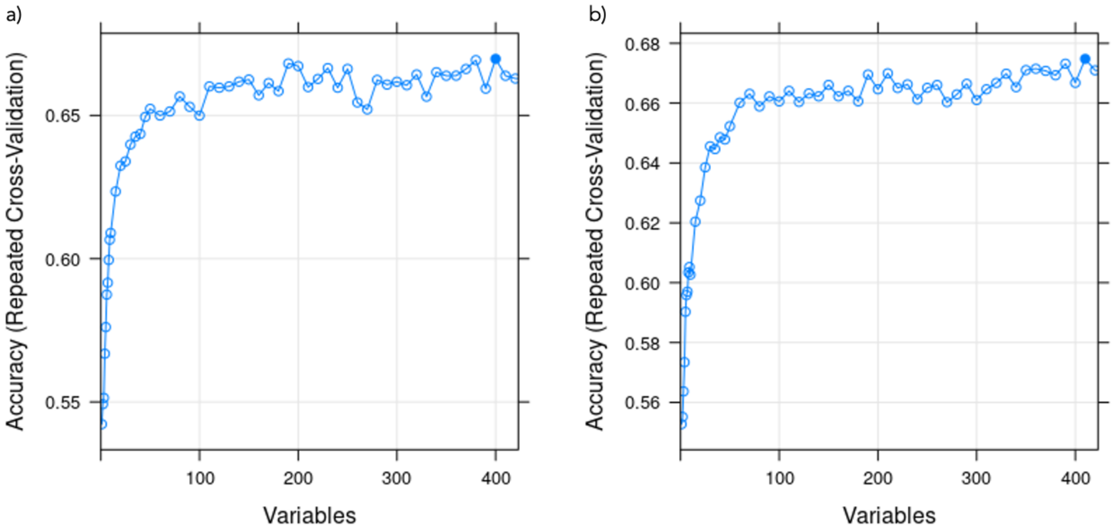

Figure S10: Kaplan-Meier survival curves for BRCA subtypes by considering various combinations of different omics types; Methylation, RNASeq, miRNA and Copy number.

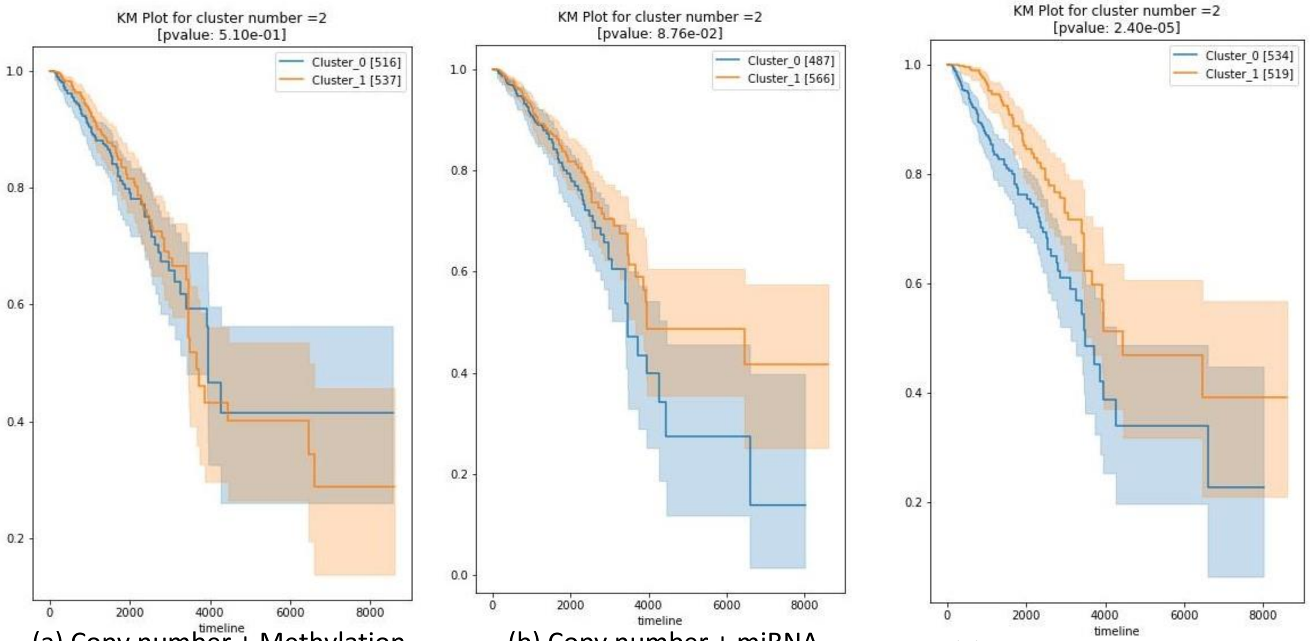

(a) Copy number + Methylation (b) Copy number + miRNA (c) Copy number + RNASeq

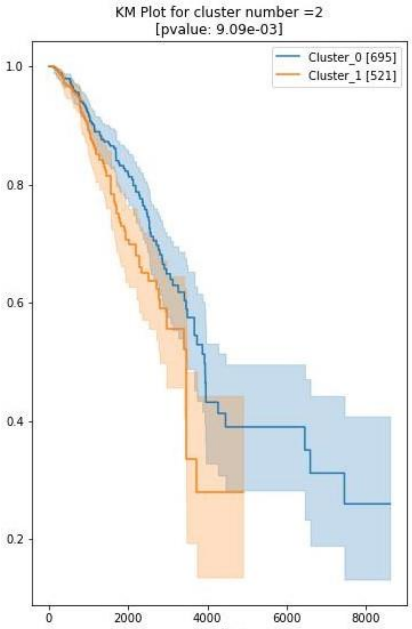

(d) Methylation + miRNA

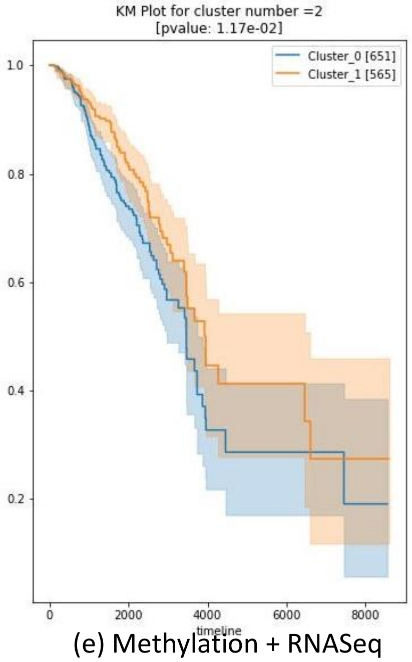

(e) Methylation + RNASeq

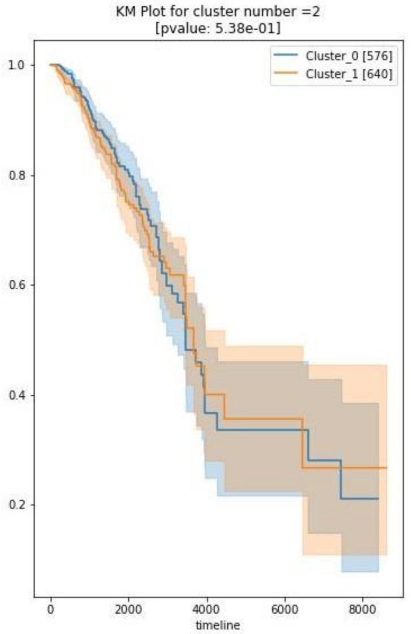

(f) RNASeq + miRNA

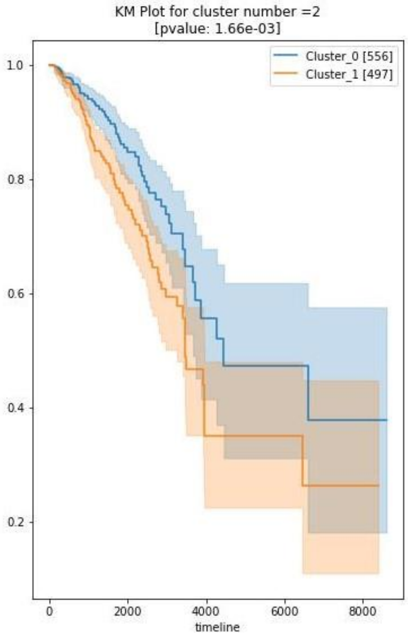

(g) Copy number + Methylation +miRNA

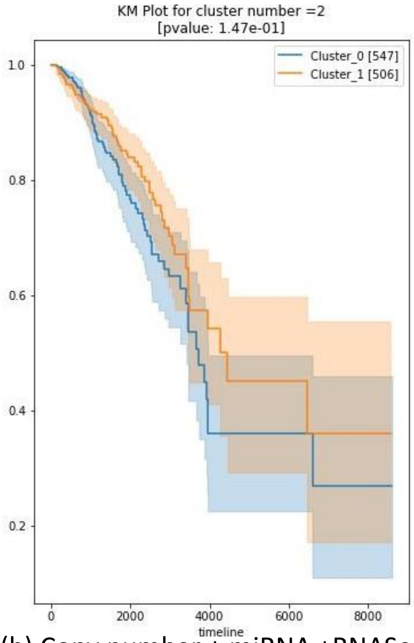

(h) Copy number + miRNA +RNASeq

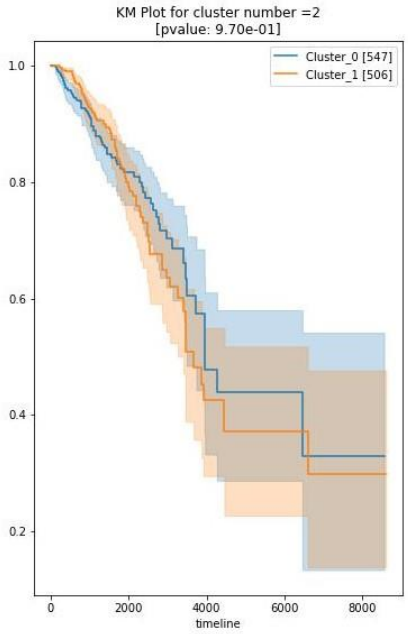

(i) Copy number + RNASeq + Methylation

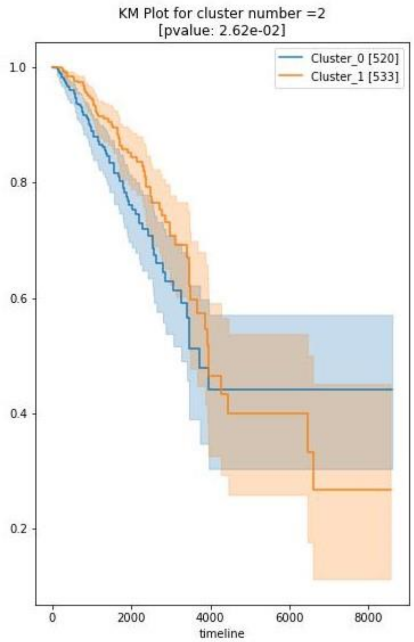

(i) Copy number + RNASeq + miRNA + RNASeq

Figure S11: Kaplan-Meier survival curves for LUAD subtypes by considering various combinations of different omics types; Methylation, RNASeq, miRNA and Copy number.

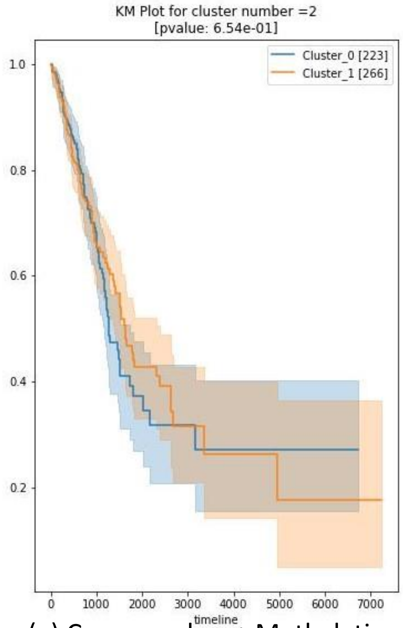

(a) Copy number + Methylation

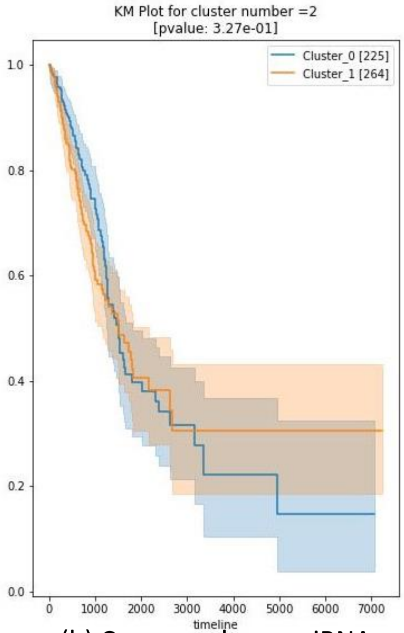

(b) Copy number + miRNA

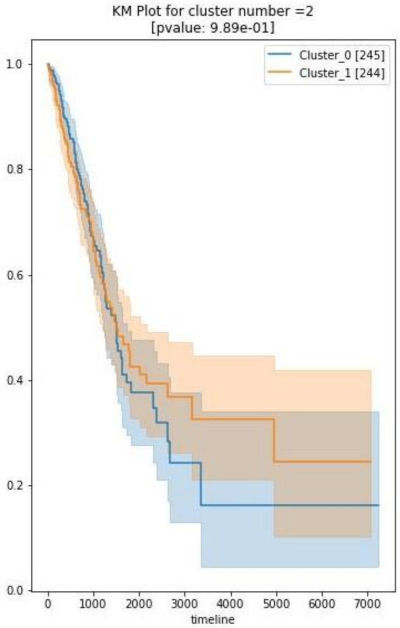

(c) Copy number + RNASeq

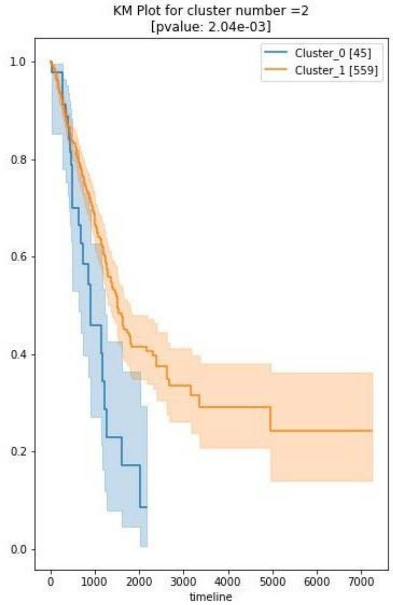

(d) Methylation + miRNA

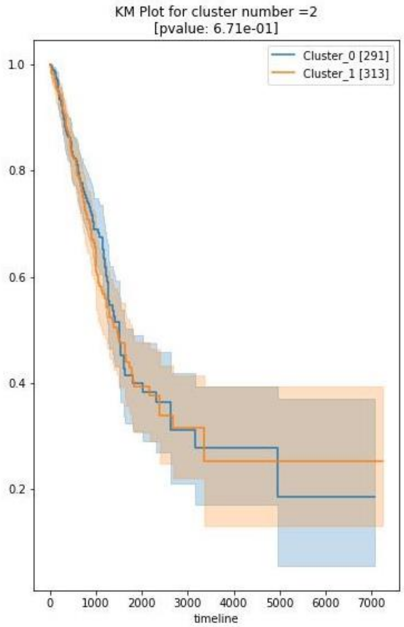

(e) Methylation + RNASeq

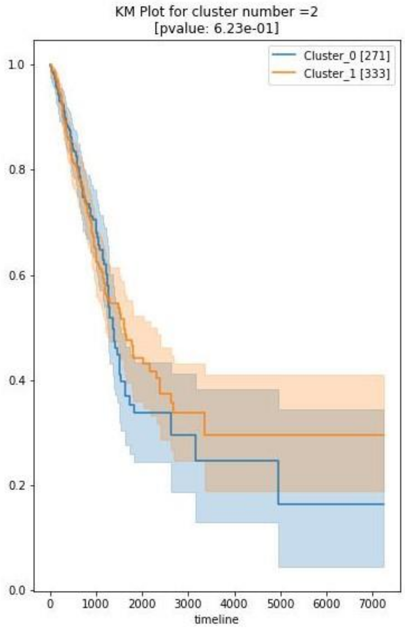

(f) RNASeq + miRNA

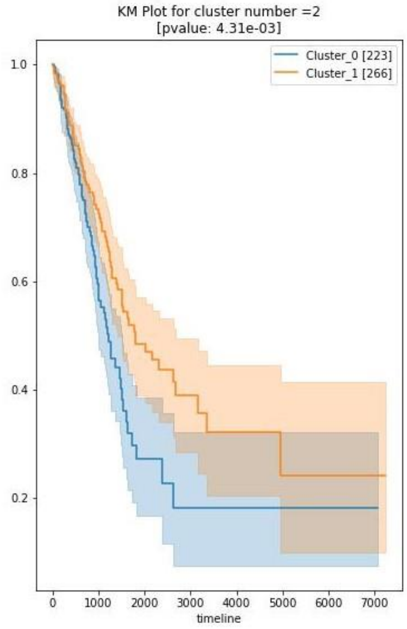

(g) Copy number + Methylation +miRNA

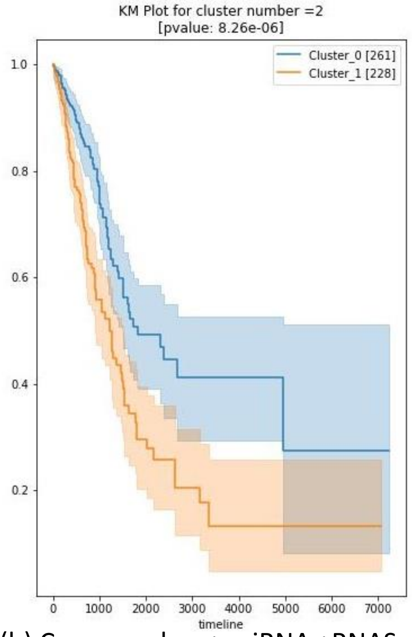

(h) Copy number + miRNA +RNASeq

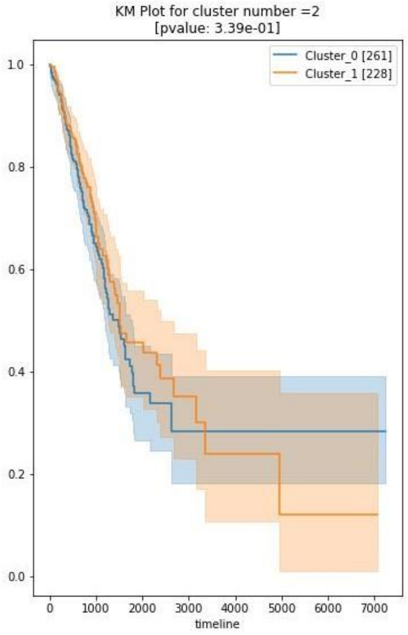

(i) Copy number + RNASeq + Methylation

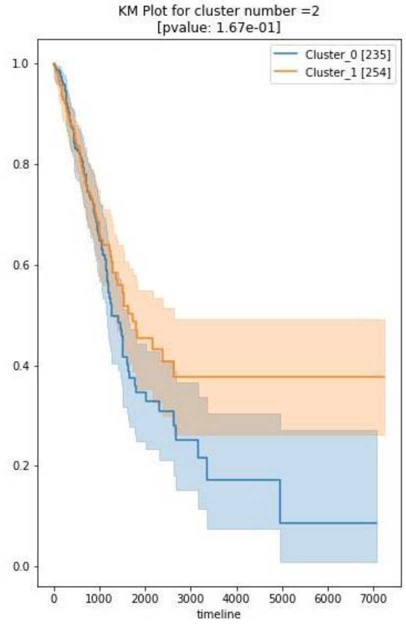

(i) Copy number + RNASeq + miRNA + RNASeq

Figure S12: Kaplan-Meier survival curves for LIHC subtypes by considering various combinations of different omics types; Methylation, RNASeq, miRNA and Copy number.

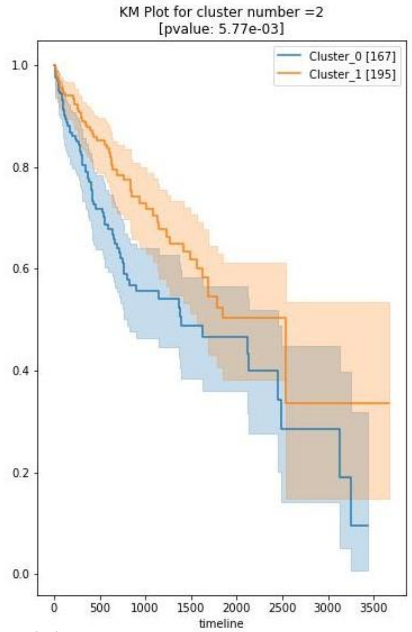

(a) Copy number + Methylation

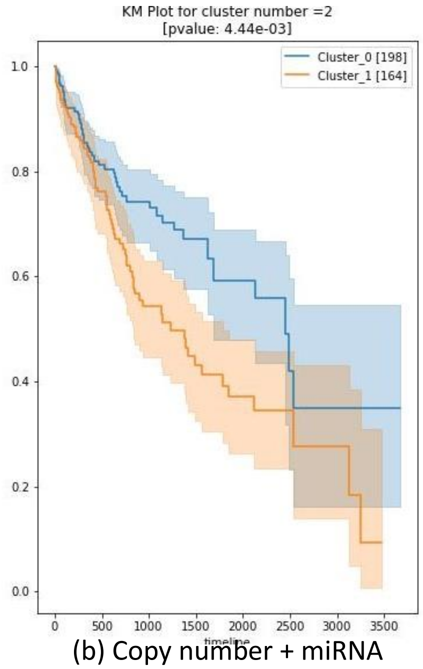

(b) Copy number + miRNA

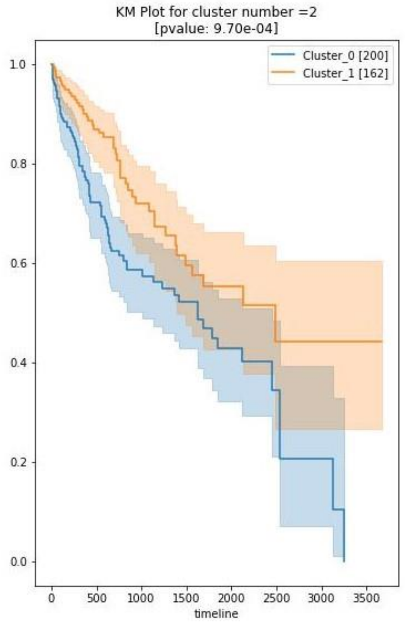

(c) Copy number + RNASeq

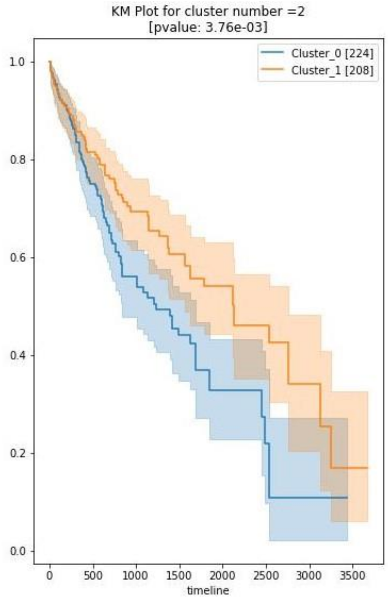

(d) Methylation + miRNA

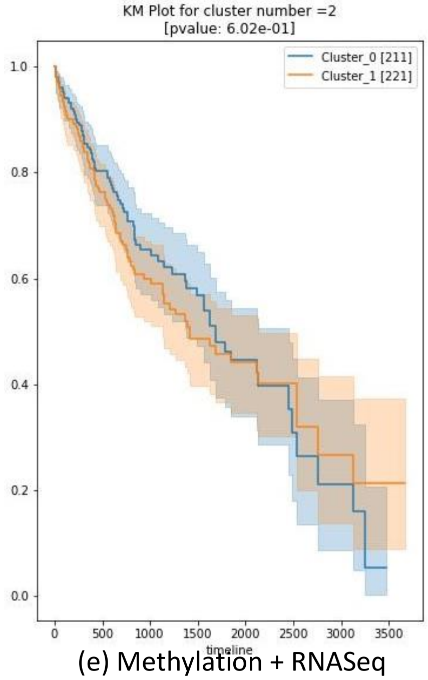

(e) Methylation + RNASeq

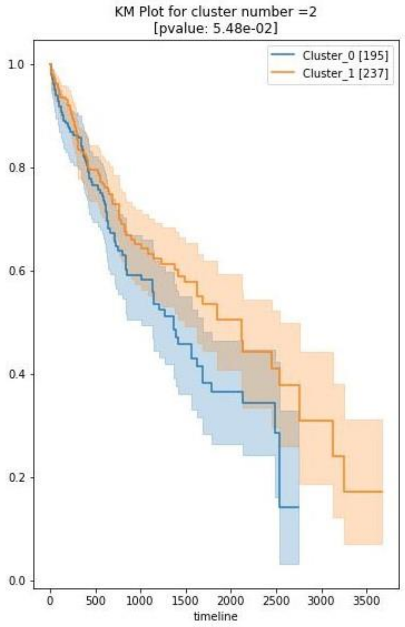

(f) RNASeq + miRNA

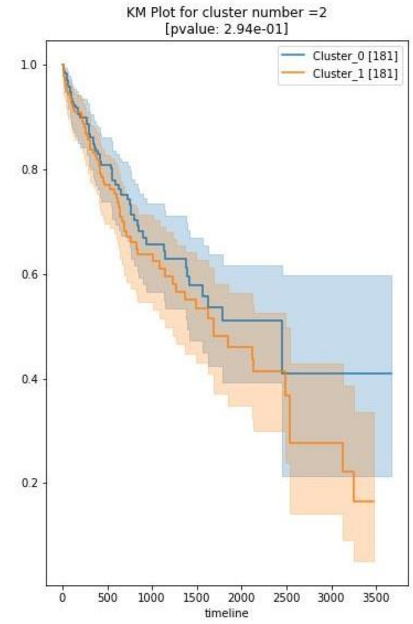

(g) Copy number + Methylation +miRNA

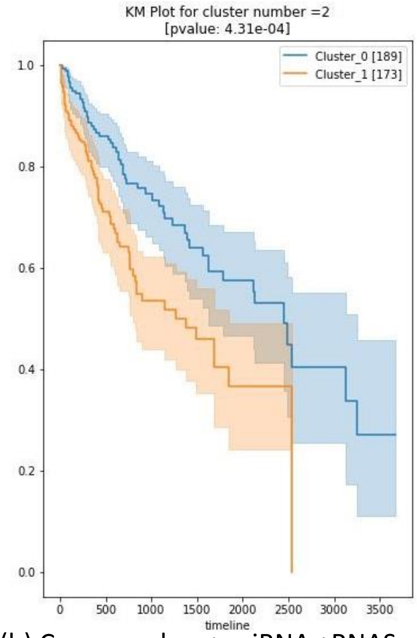

(h) Copy number + miRNA +RNASeq

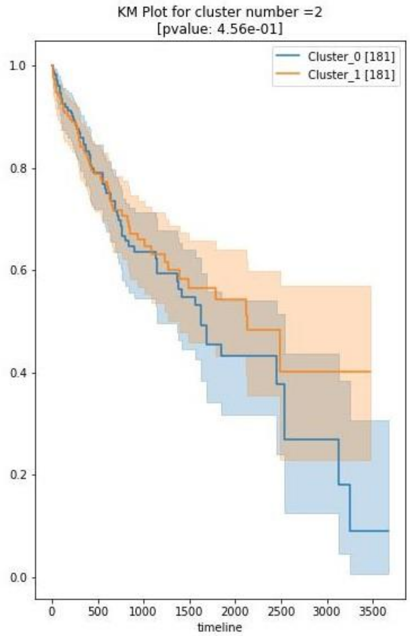

(i) Copy number + RNASeq + Methylation

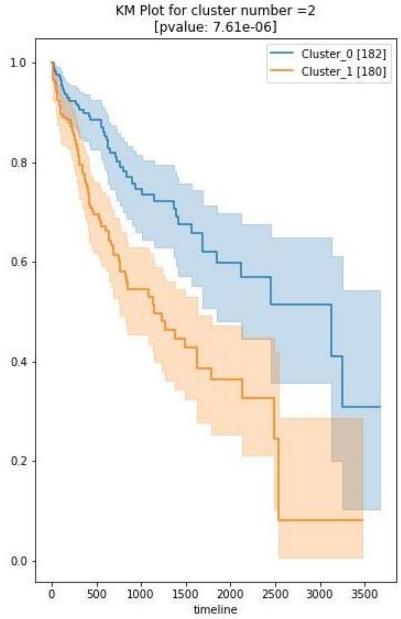

(i) Copy number + RNASeq + miRNA + RNASeq

Supplementary Tables

Table S1: Hyperparameter settings for the DGCCA algorithm

Epochs	20
Latent dimension	100
Train batch size	64
Validation batch size	64
L1 loss	0.001
L2 loss	0.0001
Learning rate	1.00e-06
Architecture	[[N,500],[500,100]], N is the number of the features for the corresponding omics(view) data
Weights between views	1:1:1
